# Dose-dependent effects of dietary quercetin on performance, egg quality, metabolic health, and antioxidant defense in laying hens: A systematic review and meta-analysis

**DOI:** 10.14202/vetworld.2026.149-164

**Published:** 2026-01-14

**Authors:** Slamet Hartanto, Heru Ponco Wardono, Heri Kurnianto, Franciscus Rudi Prasetyo Hantoro, Amrih Prasetyo, Bambang Haryanto, Rini Nur Hayati, Dini Dwi Ludfiani, Rita Purwasih, Aan Andri Yano, Joko Sujiwo, Aera Jang, Sugiharto Sugiharto

**Affiliations:** 1 Research Center for Animal Husbandry, National Research and Innovation Agency, Bogor, West Java 16911, Indonesia; 2 Research Center for Veterinary Science, National Research and Innovation Agency, Bogor, West Java 16911, Indonesia; 3 Research Center for Sustainable Production System and Life Cycle Assessment, National Research and Innovation Agency, Serpong, Banten 15314, Indonesia; 4 Faculty of Animal and Agricultural Sciences, Diponegoro University, Semarang, Central Java 50275, Indonesia; 5 Department of Agroindustry, Subang State Polytechnic, Subang, West Java 41285, Indonesia; 6 Vocational School, Sebelas Maret University, Surakarta, Central Java 57126, Indonesia; 7 Department of Animal Production, Faculty of Animal Science, Gadjah Mada University, Sleman, Special Region of Yogyakarta 55281, Indonesia; 8 Department of Applied Animal Science, Kangwon National University, Chuncheon 24341, Republic of Korea

**Keywords:** antioxidant defense, egg quality, laying hens, meta-analysis, oxidative stress, phytogenic feed additive, poultry performance, quercetin supplementation

## Abstract

**Background and Aim:**

Quercetin is a plant-derived flavonoid known for its antioxidant and metabolic regulatory properties. Many studies have assessed its effects on laying hen performance, egg quality, blood metabolites, and oxidative status; however, the results have been inconsistent, mainly due to differences in dosage, duration, hen age, and quercetin form. This meta-analysis aims to quantitatively synthesize the available evidence and examine the dose–response relationships of dietary quercetin supplementation on productive performance, egg quality traits, blood metabolites, and antioxidant defenses in laying hens.

**Materials and Methods:**

A systematic literature search of Scopus and Web of Science identified 27 eligible studies published in English. Effect sizes were calculated as mean differences (MDs) using a restricted maximum likelihood random-effects model. Subgroup and meta-regression analyses were conducted to evaluate how quercetin dose, treatment duration, initial hen age, and quercetin form (extract vs. plant powder) influenced the outcomes. Heterogeneity was assessed with the I² statistic, and publication bias was examined using funnel plots and Egger’s regression test.

**Results:**

Dietary quercetin significantly improved laying rate (LR) (MD = 2.82%), egg weight (MD = 1.21 g), Haugh unit (MD = 1.84%), shell thickness (MD = 0.014 mm), and yolk color (MD = 0.53), while reducing the feed-to-egg ratio (FER) (MD = −0.15) (p < 0.05). Quercetin supplementation also decreased serum glutamate pyruvate transaminase (SGPT), glucose, total cholesterol, and malondialdehyde levels, while increasing high-density lipoprotein and superoxide dismutase (SOD) concentrations (p < 0.05). Meta-regression revealed linear dose-dependent reductions in SGPT, glucose, and total cholesterol, whereas LR, FER, and SOD activity showed quadratic responses. Optimal responses occurred at quercetin doses of approximately 400–600 mg/kg. Treatment duration, hen age, and quercetin form further influenced several outcomes.

**Conclusion:**

Dietary quercetin effectively boosts productivity, egg quality, metabolic health, and antioxidant defense in laying hens in a dose-dependent way. Supplementing at 400–600 mg/kg seems optimal for maximizing laying performance and antioxidant levels, supporting quercetin as a promising phytogenic feed additive for sustainable poultry farming.

## INTRODUCTION

Poultry production is essential for global food security and human nutrition by efficiently transforming agri-food byproducts into high-quality meat and eggs through short production cycles [1]. However, poultry are highly susceptible to infectious diseases and environmental stressors, which can negatively impact productivity and product quality [2]. Reduced productivity threatens the sustainability of the poultry industry. Nutritional management is therefore seen as a key strategy to improve productivity and reduce disease-related losses in poultry systems [3]. In this context, natural bioactive compounds have gained increasing interest as functional dietary interventions to boost hen health and production performance [4].

Quercetin is a plant-derived flavonoid widely recognized for its powerful antioxidant, anti-inflammatory, and metabolic regulatory properties [5]. Evidence from animal studies shows that dietary quercetin lowers malondialdehyde (MDA) levels and strengthens antioxidant defense mechanisms [6]. As a result, quercetin has been extensively studied as a dietary supplement to enhance performance and egg quality in laying hens. Several studies report that quercetin supplementation positively influences key production parameters, including feed intake (FI) [7–9], laying rate (LR) [10, 11, 12–15], and feed-to-egg ratio (FER) [16–18]. Improvements have also been observed in egg quality traits such as Haugh unit (HU) [16, 17, 19], shell thickness (ST) [16, 20, 21], egg weight (EW) [15, 17, 21], and yolk color (YC) [17, 18, 20]. Furthermore, quercetin administration has been shown to decrease MDA levels [21–23] and improve antioxidant status, indicated by increased catalase (CAT) [12, 14, 21] and superoxide dismutase (SOD) activities [17, 21, 22]. Positive effects on blood metabolites, including reductions in serum glutamate pyruvate transaminase (SGPT) [10], glucose [10, 12], and total cholesterol [12, 23, 24], along with increases in high-density lipoprotein (HDL) and decreases in low-density lipoprotein (LDL) levels [12, 24], have also been reported. Additionally, quercetin has been linked to improved albumen quality [25], increased calcium deposition in reproductive tissues [26], and hepatoprotective effects in hens [27].

Despite these positive findings, several studies have reported no significant effects of quercetin on FI [28–30], LR [21, 31, 32], or FER [23, 28, 31]. Similarly, quercetin supplementation has shown no impact on EW [14, 29, 32], HU [28, 31, 33], ST [28, 31, 33], or YC [24, 28, 33] in some studies. Null effects have also been seen for blood metabolites, including glucose [18, 19], total cholesterol [8, 18, 22], HDL [18, 19], and LDL [18], as well as for antioxidant enzymes like SOD [18] and CAT [17, 22]. These differences are mainly due to variations in quercetin dosage, supplementation duration, and the hens’ age at the start of treatment. Therefore, a thorough statistical analysis is needed to determine the best application parameters for quercetin in laying hens.

Meta-analysis offers a strong quantitative method for combining data from studies with varied results, allowing for more dependable and evidence-based conclusions to be drawn [34, 35].

Although quercetin has been extensively studied as a phytogenic feed additive in laying hens, the existing research shows significant inconsistencies and unresolved questions. Some studies report beneficial effects, while others find negligible impacts on productive performance, egg quality, blood metabolites, and antioxidant status. These conflicting results are mainly due to wide variations in experimental conditions, such as quercetin dose, duration of supplementation, hen age, genetic background, and the form of quercetin used (plant powder versus extract). Therefore, it remains unclear whether the observed effects are due to quercetin itself or to differences in study design and biological context.

Importantly, most previous studies have examined quercetin’s effects in isolation within narrow experimental conditions and limited dose ranges, making it challenging to establish an evidence-based optimal supplementation strategy. The lack of a comprehensive quantitative synthesis has impeded the identification of dose–response relationships and threshold levels beyond which quercetin may become ineffective or less efficient. Additionally, while several studies have explored productive and egg quality traits, fewer have simultaneously incorporated metabolic health indicators (e.g., glucose, lipid profile, liver enzymes) and oxidative stress biomarkers, despite their vital roles in maintaining long-term productivity and hen welfare.

Another significant gap is the limited understanding of how key moderators, such as hen age, treatment duration, and quercetin form, interact with supplementation outcomes. Without considering these moderators, conclusions drawn from individual studies remain fragmented and are hard to generalize. To date, no meta-analytical study has systematically assessed these sources of heterogeneity to provide reliable, quantitative evidence guiding the practical use of quercetin in laying hen nutrition. Addressing these gaps is crucial for developing science-based recommendations that support sustainable poultry production and decrease reliance on synthetic growth promoters.

Given the limitations mentioned above, the current study aimed to perform a thorough systematic review and meta-analysis to quantitatively assess the effects of dietary quercetin supplementation on performance, egg production, egg quality, blood metabolites, oxidative stress markers, and antioxidant defense systems in laying hens. Specifically, this study intended to (i) determine the overall strength of quercetin’s effects across various productive and physiological parameters; (ii) explore dose–response relationships and identify optimal supplementation levels; and (iii) evaluate the impact of key moderators, including quercetin dose, treatment duration, initial hen age, and form of quercetin, on the observed outcomes.

By integrating data from diverse studies using rigorous meta-analytical and meta-regression approaches, this research aimed to resolve inconsistencies in the literature and provide evidence-based guidance on the effective use of quercetin in laying hen diets. The findings are expected to support precision nutrition strategies, enhance productivity and metabolic health, and contribute to the development of sustainable and resilient poultry production systems.

## MATERIALS AND METHODS

### Ethical approval

Ethical approval was not needed for this study because it involved secondary analysis of published data. All included studies adhered to institutional animal care and use guidelines.

### Study period and location

The meta-analysis was carried out from October 2024 to March at the National Research and Innovation Agency (BRIN), Indonesia, in collaboration with the Faculty of Animal and Agricultural Sciences, Diponegoro University, Indonesia.

### Study protocol and reporting framework

This meta-analysis compiled evidence from previously published studies assessing the effects of dietary quercetin supplementation in laying hens. The research question was developed using the PICO framework [36], where the population (P) consisted of laying hens, the intervention (I) was quercetin supplementation, the comparator (C) was a control diet without quercetin, and the outcomes (O) included egg production and egg quality traits. The study protocol adhered to the Preferred Reporting Items for Systematic Reviews and Meta-Analyses (PRISMA) guidelines, with reporting aligned to the PRISMA 2020 statement (https://doi.org/10.5281/ zenodo.17718238) [37]. Formal protocol registration was not performed because the study began before registration was required.

### Literature search strategy and data sources

A comprehensive literature search strategy is summarized in Table 1. Searches were conducted in the Scopus and Web of Science databases on October 7, 2024. The Scopus search string was: TITLE-ABS-KEY (hen*) AND TITLE-ABS-KEY (quercetin) AND TITLE-ABS-KEY (egg*). For Web of Science, the query was: TS = (hen*) AND TS = (quercetin) AND TS = (egg*). No restrictions were applied regarding publication year. Only original research articles were included, while reviews, preprints, and conference proceedings were excluded. Articles not published in English were also excluded. The initial search yielded 116 records, comprising 58 articles from each database.

**Table 1:** Keyword combinations for the literature search.

Databases	Keywords	Studies
Scopus	TITLE-ABS-KEY (hen*) AND TITLE-ABS-KEY (quercetin) AND TITLE-ABS-KEY (egg*)	58
Web of Science	#1 TS=(hen*)	
	#2 TS=(quercetin)	
	#3 TS=(egg*)	
	#1 AND #2 AND #3	58

### Eligibility criteria

Study eligibility was determined based on the PICO framework. Studies were included if they met these criteria: (1) full-text available; (2) investigated quercetin supplementation in laying hens; (3) assessed production performance, egg quality, antioxidant status, and/or blood metabolite parameters; (4) included a control group; and (5) reported measures of variability, such as confidence intervals (CI), standard errors (SE), or standard deviations (SD). Studies using mixed antioxidant formulations (for example, quercetin combined with vitamin E or other antioxidants) were excluded. When studies reported multiple quercetin doses, each dose was considered an independent comparison to evaluate dose-dependent effects.

### Study screening and selection

**Figure 1:**
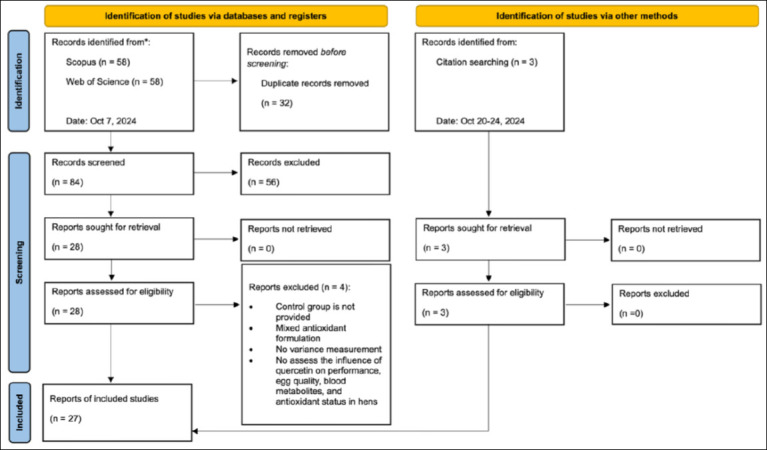
PRISMA workflow for the literature strategy.

The study selection process followed the PRISMA flow diagram (Figure-1). Zotero [38] and Microsoft Excel version 16.91 [39] were used to identify and remove duplicate records and to support title and abstract screening. Six reviewers (SH, HK, HPW, FRPH, AP, and BH) independently screened titles and abstracts for eligibility. Full-text articles were independently assessed by two reviewers (SH and HK), with discrepancies resolved by a third reviewer (HPW). After duplicate removal, 84 records remained. Following title and abstract screening, 28 studies were retained for full-text evaluation, of which 24 met the inclusion criteria. Manual screening of reference lists between October 20 and October 24, 2024, identified three additional eligible studies. In total, 27 studies were included in the final meta-analysis.

### Data extraction and standardization

The characteristics of the included studies are shown in Table 2 [7–33]. Data collected included performance indicators ([FI] and FER), egg production parameters (LR and EW), egg quality traits (ST, YC, EW, and HU), blood metabolites (SGPT, glucose, total cholesterol, HDL, and LDL), oxidative stress markers (malondialdehyde [MDA]), and antioxidant enzyme activities (catalase [CAT] and SOD). Eight reviewers independently extracted data in duplicate using a standardized extraction form. Any disagreements were resolved through discussion, with a third reviewer (SH) consulted if consensus was not reached.

**Table 2:** Characteristics of the included studies.

Study	Year	Country	Breed	N	Initial age (weeks)	(weeks)	Form	Dose (mg/kg)
Liu *et al.* [7]	2013	China	Hessian	240	≤50	5-8	Extract	0, 200, 400, and 600
Simitzis *et al.* [8]	2018	Greece	Lohmann Brown-Classic	192	>50	≤4	Extract	0, 200, 400, and 800
Amevor *et al.* [9]	2021	China	Tianfu	400	>50	>8	Extract	0, 400
Ahmad *et al*. [10]	2018	Pakistan	HyLine W36	200	≤50	5-8	Plant powder	0.42, 16.57, 32.88, 48.88
Yang *et al.* [11]	2016	China	Hessian	240	≤50	5-8	Extract	0, 200, 400, and 600
El-Saadany *et al.* [12]	2022	Egypt	Mandarah	200	≤50	>8	Extract	0, 300, 600, and 1200
Shen *et al.* [13]	2021	China	Wenchang and Yellow Rugao	350	≤50	5-8	Plant powder	0, 594.5, 1189, 1664.6, 2378
Liu *et al*. [14]	2023	China	Hyline Brown	360	>50	>8	Extract	0, 500
Fu *et al*. [15]	2024	China	Hy-Line Brown	2,360	>50	≤4	Extract	0, 500
Ahmad *et al*. [16]	2017	Pakistan	HyLine W36	200	≤50	5-8	Plant powder	0.48, 7.98, 15.58, and 22.85
Lin *et al.* [17]	2017	Taiwan	Hendrix	96	≤50	>8	Plant powder	0, 22, 44, 88
Su *et al*. [18]	2020	Taiwan	ISA Brown	80	≤50	>8	Plant powder	0, 48.8, 97.6, and 195.2
Wei *et al.* [19]	2023	China	Hy-Line Brown	240	≤50	>8	Extract	0, 300
Abid *et al.* [20]	2019	Iraq	Isa Brown	120	≤50	>8	Extract	0, 400, 800, and 1200
Cao *et al.* [21]	2024	China	Tianfu	400	≤50	5-8	Extract	0, 400
Amevor *et al*. [22]	2021	China	Tianfu	400	≤50	>8	Extract	0, 400
Iskender *et al.* [23]	2016	Turkey	Lohmann White	96	≤50	5-8	Extract	0, 500
Liu *et al*. [24]	2023	China	Hyline Brown	360	>50	>8	Extract	0, 500
Damaziak *et al.* [25]	2017	Poland	ISA Brown	216	≤50	>8	Extract	0, 6
Huang *et al*. [26]	2022	China	Filing	270	>50	5-8	Extract	0, 30, 60
Abid *et al.* [27]	2019	Iraq	Isa Brown	120	≤50	>8	Extract	0, 400, 800, and 1200
Whiting *et al.* [28]	2022	UK	Hy-Line Brown	80	≤50	≤4	Extract	0, 1275
Amevor *et al*. [29]	2022	China	Tianfu	400	>50	>8	Extract	0, 400
Amevor *et al.* [30]	2022	China	Tianfu	400	>50	>8	Extract	0, 400
Liu *et al*. [31]	2014	China	Hessian	240	≤50	5-8	Extract	0, 200, 400, and 600
Iskender *et al.* [32]	2017	Turkey	Lohmann White	96	≤50	5-8	Extract	0, 500
Ying *et al*. [33]	2016	China	Hessian	240	≤50	5-8	Extract	0, 200, 400, and 600

N = Number of birds

Mean values and SDs were obtained or calculated for meta-analysis. When SDs were not directly reported, they were computed using the following formulas: (1) SD = SE × √N, where N is the number of replicates; and (2) SD = √N × (upper CI − lower CI)/3.92, which corresponds to a 95% CI. For small sample sizes (<60), the constant 3.92 was replaced with the appropriate t-distribution value based on degrees of freedom [40].

Graphical data were digitized using WebPlotDigitizer software [41]. Reference axes were calibrated before extracting mean values and corresponding CI limits. Extracted data were transferred to Microsoft Excel, where SDs or SEs were calculated and cross-checked against original sources. All measurement units were standardized before analysis.

### Risk of bias assessment

Two reviewers (HPW and HK) independently assessed the risk of bias using the SYRCLE risk of bias tool for animal studies [42]. The evaluation covered domains such as selection bias, performance bias, detection bias, attrition bias, reporting bias, and other possible biases. Each domain was rated as low, unclear, or high risk based on set criteria. Disagreements were resolved through discussion or by consulting a third reviewer (SH).

### Data synthesis and statistical analysis

All statistical analyses were performed using R software version 4.2.2 [43], with the “metafor” package version 4.8-0 [44]. Subgroup analysis results were visualized with the “ggplot2” package version 3.5.2 [45] and Microsoft Excel [39]. Effect sizes were calculated as MDs along with 95% CI using a restricted maximum likelihood (REML) random-effects model [46]. REML was chosen for its robustness and reliability in situations of high heterogeneity [47]. Statistical significance was defined as p < 0.05.

Heterogeneity was evaluated using the I² statistic, with values over 50%, between 25% and 50%, and below 25% indicating high, moderate, and low heterogeneity, respectively [48]. Additionally, a Q-test p-value less than 0.10 was deemed indicative of significant heterogeneity [49].

### Subgroup and meta-regression analyses

Subgroup and meta-regression analyses were performed when three criteria were satisfied: (1) a significant overall effect size (p < 0.05); (2) considerable heterogeneity (I² > 50%, p < 0.001); and (3) adequate data availability (≥ 10 comparisons) [50]. Quercetin dose (mg/kg) was considered a continuous covariate, while initial hen age (≤ 50 vs. > 50 weeks), treatment duration (≤ 4, 5–8, and ≥ 8 weeks), and quercetin form (plant powder or extract) were treated as categorical covariates. A mixed-effects model was used for subgroup analysis [51].

### Publication bias and sensitivity analysis

Publication bias was evaluated using funnel plot visualization and Egger’s regression test [52], with p < 0.05 indicating a high risk of bias. Sensitivity analysis was conducted with a leave-one-out approach to assess the robustness of the pooled estimates. The trim-and-fill method was used to estimate the potential effect of missing studies [53].

### Data and software availability

All extracted datasets, statistical scripts, and supplementary Figures 1A–1P are publicly accessible at https://doi.org/10.5281/zenodo.17626656.

## RESULTS

### Characteristics of the included studies

**Figure 2:**
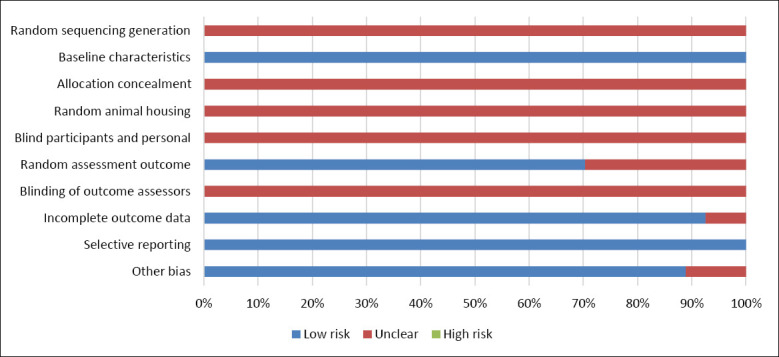
SYRCLE risk of bias.

A total of 27 eligible studies were included in this meta-analysis. The dietary quercetin doses evaluated across studies ranged from 0 to 2,378 mg/kg. Most studies involved hens with an initial age of ≤ 50 weeks (70.38%), while the remaining 29.62% included hens older than 50 weeks. Treatment duration varied significantly, with 11.11% of studies administering quercetin for less than 4 weeks, 40.74% for 5–8 weeks, and 48.15% for more than 8 weeks. Regarding formulation, quercetin was primarily provided as extract powder (81.48%), while plant powder made up 18.52% of the treatments. The nutrient composition of the basal diets used in the included studies is summarized in Table-3. Risk of bias assessment outcomes are illustrated in Figure-2, showing that all included studies had a low risk of bias regarding baseline characteristics and selective reporting domains.

**Table 3:** The nutrient composition of the basal diets in the included studies (n = 27).

Parameters	Unit	Mean	SD	Min	Max
Metabolizable energy	Kcal/kg	2,736.79	170.65	2,563	3,497
Crude protein	%	17.05	1.13	15.22	21.8
Calcium	%	3.55	0.75	0.95	4.77
Phosphorus	%	0.77	0.8	0.32	3.5

SD = Standard deviation, Min = Minimum, Max = Maximum.

### Effects of dietary quercetin on egg production and egg quality

Dietary quercetin supplementation significantly affected (p < 0.001) various performance, production, and egg quality parameters in laying hens (Table 4). Significant effects were seen for LR, FER, EW, HU, ST, and YC. In contrast, FI was not influenced by quercetin supplementation (p > 0.05). Overall, quercetin increased LR (MD = 2.819%), EW (MD = 1.209 g/unit), HU (MD = 1.838%), ST (MD = 0.014 mm), and YC (MD = 0.526), while significantly decreasing FER (MD = −0.146), indicating improved feed efficiency.

**Table 4:** Effect of dietary quercetin on egg production and quality.

Parameters	N	n	Control mean (SD)	MD (95% CI)	p-value	Heterogeneity test	

						p-value	*I^2^* (%)
Feed intake (g/d/hen)	42	3,220	99.488 (4.856)	0.902 (–0.068; 1.872)	0.068	<.001	99.72
Laying rate (%)	68	5,288	74.312 (5.393)	2.819 (1.574; 4.064)	<.001	<.001	99.46
Feed-egg-ratio	60	4,680	2.167 (0.124)	–0.146 (–0.198; –0.094)	<.001	<.001	99.87
Egg weight (g/unit)	70	4,940	60.214 (3.141)	1.209 (0.702; 1.716)	<.001	<.001	97.75
Haugh unit (%)	65	2,898	82.298 (7.580)	1.838 (0.901; 2.776)	<.001	<.001	93.55
Shell thickness (mm)	71	2,558	0.376 (0.075)	0.014 (0.007; 0.021)	<.001	<.001	89.33
Yolk color	52	2,958	7.964 (2.222)	0.526 (0.334; 0.717)	<.001	<.001	92.95

N = Number of comparisons, SD = Standard deviation, MD = Mean difference, *I^2^* = Inconsistency index.

### Effects of dietary quercetin on blood metabolite profiles

The influence of dietary quercetin on blood metabolites is shown in Table 5. Quercetin supplementation significantly impacted serum glucose, SGPT, HDL, and total cholesterol levels (p < 0.001). In contrast, LDL levels did not differ between control and quercetin-treated hens (p > 0.05). Specifically, quercetin supplementation decreased SGPT (MD = −7.009 U/L), glucose (MD = −17.589 mg/dL), and total cholesterol (MD = −20.834 mg/dL), while significantly increasing HDL levels (MD = 32.590 mg/dL).

**Table-5:** Blood metabolite parameters in control and quercetin-treated hens.

Parameters	N	n	Control mean (SD)	MD (95% CI)	p-value	Heterogeneity test	

						p-value	*I^2^* (%)
SGPT (U/L)	15	384	20.318 (1.210)	–7.009 (-9.196; –4.822)	<.001	<.001	98.95
Glucose (mg/dL)	16	400	248.060 (8.281)	–17.589 (-23.627; –11.550)	<.001	<.001	96.55
Total cholesterol (mg/dL)	30	608	150.287 (12.529)	–20.834 (-38.721; –2.948)	0.022	<.001	99.81
HDL (mg/dL)	14	184	52.068 (8.270)	32.590 (2.257; 62.924)	0.035	<.001	99.73
LDL (mg/dL)	14	184	59.593 (9.478)	–8.552 (–17.146; 0.042)	0.051	<.001	95.31

N = Number of comparisons, SD = Standard deviation, MD = Mean difference, CI = Confidence interval, *I^2^* = Inconsistency index, SGPT = Serum glutamate pyruvate transaminase, HDL = High-density lipoprotein, LDL = Low-density lipoprotein.

### Impact of quercetin on oxidative stress and antioxidant activity

Dietary quercetin supplementation significantly influenced malondialdehyde (MDA) and SOD levels in hens (p < 0.001) (Table 6). In contrast, catalase (CAT) activity was not significantly affected (p > 0.05). Quercetin treatment led to a notable decrease in MDA concentration (MD = −7.373 nmol/mL), indicating reduced lipid peroxidation, and a significant increase in SOD activity (MD = 8.114 U/mL), reflecting enhanced antioxidant defense.

**Table-6:** Comparison of MDA level and antioxidant status between control and quercetin-treated hens.

Parameters	N	n	Control mean (SD)	MD (95% CI)	p-value	Heterogeneity test	

						p-value	*I^2^* (%)
MDA, nmol/mL	16	548	19.436(2.586)	–7.373 (–12.304; –2.443)	0.003	< 0.001	99.97
SOD, U/mL	19	584	39.722(3.018)	8.114 (0.233; 15.995)	0.044	< 0.001	100
CAT, U/mL	15	512	23.363(7.976)	3.790 (–0.521; 8.102)	0.085	< 0.001	97.6

N = Number of comparisons, SD = Standard deviation, MD = Mean difference, CI = Confidence interval, *I^2^* = Inconsistency index, MDA = Malondialdehyde, SOD = superoxide dismutase, CAT = Catalase.

### Heterogeneity and subgroup analyses

All evaluated parameters showed significant heterogeneity (p < 0.001), with high inconsistency values (I² > 50%) across studies (Tables 4–6). Subgroup and meta-regression analyses identified quercetin dose as a significant moderator affecting LR (R² = 11.44%), FER (R² = 9.62%), SGPT (R² = 33.07%), glucose (R² = 29.42%), total cholesterol (R² = 28.01%), and SOD (R² = 30.71%) (Table 7). Treatment duration significantly influenced YC (R² = 9.33%), SGPT (R² = 75.29%), glucose (R² = 60.63%), and total cholesterol (R² = 44.79%). Initial hen age significantly impacted LR (R² = 5.18%), ST (R² = 24.58%), and SOD (R² = 24.89%). Additionally, the form of quercetin significantly moderated LR (R² = 4.99%), FER (R² = 9.75%), YC (R² = 16.98%), total cholesterol (R² = 26.17%), and SOD (R² = 22.11%).

**Table 7:** Moderator test of meta-regression of quercetin treatment in hens.

Parameter	Covariates	QM	d	p-value	R^2^(%)
Laying rate	Dose	9.602	2	0.008	11.44
	Duration	1.252	2	0.535	0
	Initial age	3.905	1	0.048	5.18
	Form	4.138	1	0.042	4.99
Feed-to-egg ratio	Dose	7.399	2	0.025	9.62
	Duration	2.269	2	0.322	0
	Initial age	0.3	1	0.584	0
	Form	5.9	1	0.015	9.75
Egg weight	Dose	2.293	1	0.130	2.72
	Duration	1.575	2	0.455	0.29
	Initial age	2.162	1	0.141	4.74
	Form	0.481	1	0.488	0
Haugh unit	Dose	2.848	1	0.091	2.48
	Duration	3.454	2	0.178	0
	Initial age	0.067	1	0.795	0
	Form	0.012	1	0.914	0
Shell thickness	Dose	0.078	1	0.780	0
	Duration	2.316	2	0.314	0.29
	Initial age	18.014	1	<0.001	24.58
	Form	1.691	1	0.193	1.78
Yolk color	Dose	1.565	1	0.211	2.79
	Duration	6.595	2	0.037	9.33
	Initial age	0.075	1	0.784	0
	Form	6.936	1	0.008	16.98
SGPT	Dose	7.741	1	0.005	33.07
	Duration	42.522	2	<0.001	75.29
	Initial age	NA	NA	NA	NA
	Form	NA	NA	NA	NA
Glucose	Dose	4.995	1	0.025	29.42
	Duration	20.545	2	<0.001	60.63
	Initial age	NA	NA	NA	NA
	Form	0	1	0.994	0
Total cholesterol	Dose	11.635	1	<0.001	28.01
	Duration	22.999	2	<0.001	44.79
	Initial age	0.038	1	0.846	0
	Form	10.426	1	0.001	26.17
HDL	Dose	0.632	1	0.426	0
	Duration	0.003	1	0.959	0
	Initial age	0.003	1	0.959	0
	Form	1.117	1	0.291	0.87
MDA	Dose	0.034	1	0.853	0
	Duration	1.295	2	0.523	0
	Initial age	0.449	1	0.503	0
	Form	0.621	1	0.431	0
SOD	Dose	8.706	2	0.013	30.71
	Duration	0.605	2	0.739	0
	Initial age	8.989	1	0.003	24.89
	Form	5.328	1	0.021	22.11

QM = Coefficient of moderators, d = Degree of freedom, R^2^ = Heterogeneity accounted for by covariate, NA = Not available, SGPT = Serum glutamate pyruvate transaminase, HDL = High-density lipoprotein, MDA = Malondialdehyde, SOD = superoxide dismutase.

### Dose–response relationships of quercetin supplementation

**Figure 3:**
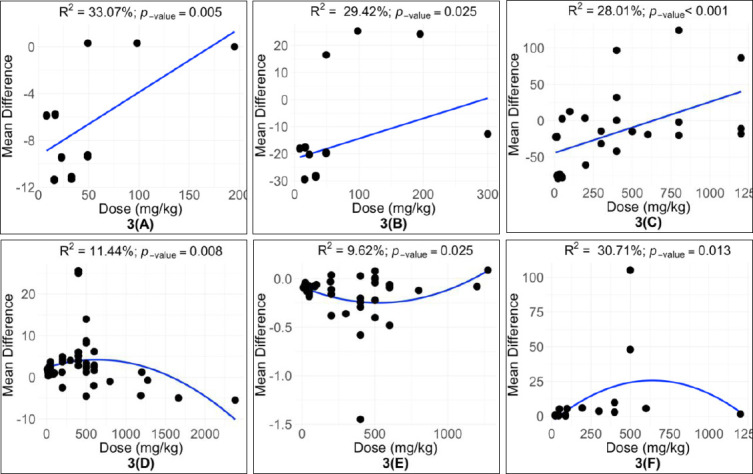
Subgroup analysis for the effect of quercetin dose on (A) serum glutamate pyruvate transaminase (U/L), (B) glucose (mg/ dL), (C) total cholesterol (mg/ dL), (D) laying rate (%), (E) feed-to-egg ratio, and (F) superoxide dismutase (U/mL) levels in hens.

Meta-regression analysis showed that the quercetin dose had a linear effect on SGPT, glucose, and total cholesterol levels (Table 8; Figures 3A–C). In contrast, LR, FER, and SOD showed quadratic responses to increasing quercetin doses. The highest LR was observed at 600 mg/kg (Figure 3D), while the optimal FER occurred between 400–600 mg/kg (Figure 3E). The highest SOD activity was recorded at approximately 500 mg/kg of dietary quercetin (Figure 3F).

**Table 8:** Subgroup analysis: regression model for the relationship between quercetin dose and hen parameters.

Equation	Model	Intercept			*X*			χ²			RMSE	AIC	BIC
		
		Coefficient	SEM	p-value	Coefficient	SEM	p-value	Coefficient	SEM	p-value			
Laying rate	Q	2.302	0.857	0.007	6.029 × 10^-3^	3.283 × 10^-3^	0.066	–4.730 × 10^-6^	1.740 × 10^-6^	0.006	4.602	399.027	407.725
Feed-to-egg ratio	Q	–0.084	0.037	0.023	–6.23 × 10^-4^	2.302 × 10^-4^	0.007	0.590 × 10^-6^	0.230 × 10^-6^	0.009	0.196	–14.211	–6.038
SGPT	L	–9.314	1.237	<.001	0.054	0.02	0.005	-	-	-	3.283	75.755	77.45
Glucose	L	–21.846	2.843	<.001	0.075	0.033	0.025	-	-	-	16.42	119.099	121.016
Total cholesterol	L	–44.353	10.297	<.001	0.07	0.021	<.001	-	-	-	43.3286	297.026	301.023
SOD	Q	–4.265	5.287	0.42	9.367 × 10^-2^	3.224 × 10^-2^	0.003	–7.314 × 10^-5^	2.886 × 10^-6^	0.011	20.86821	158.435	160.934

L = Linear model, Q = Quadratic model, SEM = Standard error of the mean, *X*= Linear term power of the variable, χ² = Quadratic term power of the variable, RMSE = Root mean square error, AIC = Akaike information criterion, BIC = Bayesian information criterion, SGPT = Serum glutamate pyruvate transaminase, SOD = superoxide dismutase.

### Effects of treatment duration

**Figure 4:**
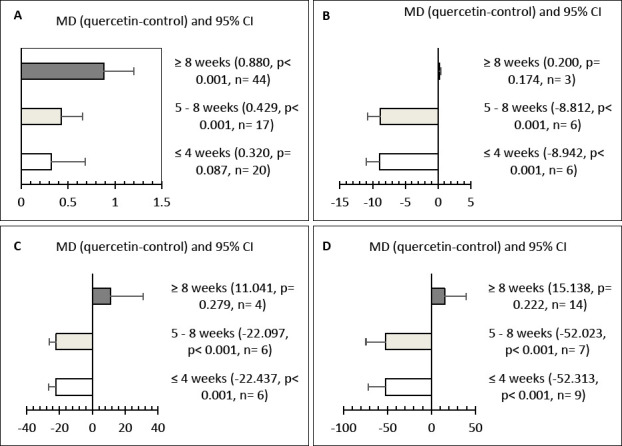
Effect of treatment duration on (A) yolk color, (B) serum glutamate pyruvate transaminase (U/L), (C) glucose (mg/dL), and (D) total cholesterol (mg/dL) in hens. CI = Confidence interval.

Treatment durations of 5–8 weeks and over 8 weeks significantly improved YC (p < 0.05) (Figure 4A), while supplementation for less than 4 weeks had no significant impact. Short-term (<4 weeks) and medium-term (5–8 weeks) supplementation significantly decreased SGPT, glucose, and total cholesterol levels (p < 0.001) (Figures 4B–D). However, these blood metabolite improvements were not observed when supplementation was extended beyond 8 weeks.

### Influence of initial hen age

**Figure 5:**
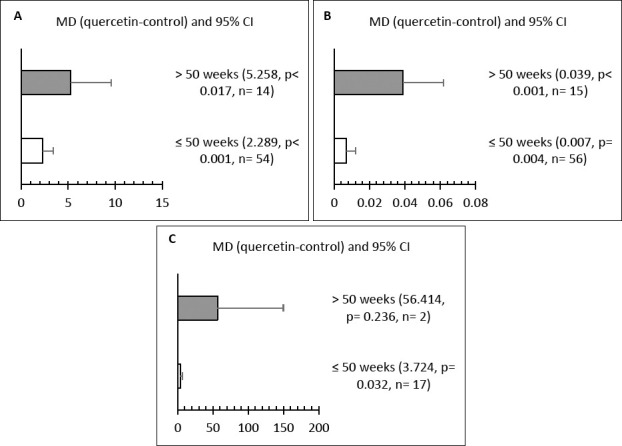
Subgroup analysis of the effect of initial age on (A) laying rate (%), (B) shell thickness (mm), and (C) superoxide dismutase (SOD) (U/mL) in hens.

Subgroup analysis based on initial hen age showed significant effects on LR, ST, and SOD levels (Figures 5A–C). Hens older than 50 weeks had higher LR and ST compared to both the control and the ≤ 50-week groups (p < 0.05). Additionally, quercetin supplementation in hens aged ≤ 50 weeks significantly increased LR, ST, and SOD activity (p < 0.05).

### Effect of quercetin form

**Figure 6:**
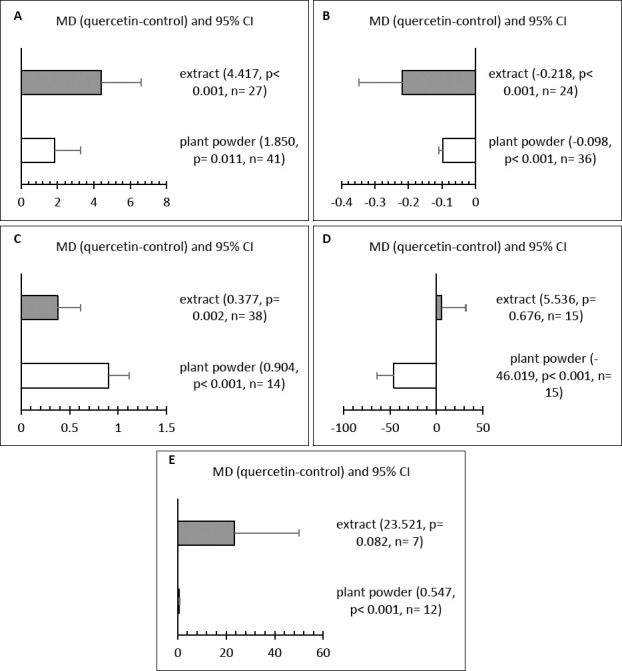
Subgroup analysis for the effect of quercetin form on (A) laying rate (%), (B) feed-to-egg ratio, (C) yolk color, (D) total cholesterol (mg/ dL), and (E) superoxide dismutase (U/mL) in hens.

The form of quercetin significantly affected LR, FER, YC, total cholesterol, and SOD levels (Figures 6A–E). Quercetin provided as an extract powder resulted in the highest LR and the lowest FER (p < 0.05), indicating better productivity and feed efficiency. In contrast, plant powder supplementation produced the highest YC scores and the lowest total cholesterol levels. Extract powder had no notable effect on SOD activity, while plant powder significantly increased SOD levels.

### Publication bias and sensitivity analysis

Assessment of publication bias was conducted using funnel plots and Egger’s regression test (Suppl. Figures 1A–P; https://doi.org/10.5281/zenodo.17626656). A low risk of publication bias (p > 0.05) was found for LR, FER, EW, HU, ST, YC, SGPT, HDL, LDL, and CAT. Conversely, significant publication bias (p < 0.05) was observed for glucose, total cholesterol, MDA, and SOD outcomes. Leave-one-out sensitivity analysis verified the robustness of the results, as removing individual studies did not significantly change the pooled effect estimates.

## DISCUSSION

### Effects of quercetin on productive performance and egg quality

Dietary quercetin supplementation significantly enhanced productivity and egg quality in laying hens, as shown by improvements in FER, LR, and egg quality traits such as EW, HU, ST, and YC. These results align with those reported by Liu *et al*. [7], who found that quercetin supplementation positively affected LR, EW, FER, HU, and ST in hens. Additionally, the better YC scores in quercetin-fed hens were accompanied by improvements in LR, FER, HU, and ST. Similar gains were reported by Ahmad *et al*. [10], who observed that quercetin-rich mulberry leaf supplementation significantly increased LR, HU, and ST. The improvements in productivity and egg quality may be due to quercetin’s effects on blood metabolites, antioxidant capacity, and reproductive physiology in laying hens.

### Modulation of blood metabolites and hepatic function

Quercetin supplementation had significant effects on blood metabolite profiles, especially lipid metabolism. Serum total cholesterol and HDL concentrations are important markers of lipid metabolic health in poultry [54]. In this study, quercetin notably lowered total cholesterol levels and increased HDL concentrations. These findings align with those of El-Saadany *et al*. [12] and Liu *et al*. [24], who observed decreased serum total cholesterol and higher HDL levels in hens supplemented with quercetin. Since hepatic lipoprotein synthesis is crucial for follicular development in laying hens [55], improved serum lipid profiles indicate better liver function and reproductive performance. Lower total cholesterol and elevated HDL also suggest a decreased risk of fatty liver syndrome [56].

Mechanistically, quercetin has been shown to promote cholesterol homeostasis by enhancing the selective uptake of HDL-derived lipids [57]. Maintaining cholesterol balance is essential for liver health, as dysregulation can cause intrahepatic lipid buildup and subsequent liver injury [58]. The significant decrease in SGPT observed in this analysis further supports the hepatoprotective role of quercetin. Previous research has similarly indicated reduced SGPT activity following flavonoid supplementation, which suggests improved liver health [59]. Notably, Amevor *et al*. [22] reported that quercetin alleviated hepatic steatosis in aging hens. Overall, these findings suggest that quercetin improves liver function and lipid metabolism, thereby enhancing egg production and quality.

### Enhancement of antioxidant defense and oxidative stress mitigation

Dietary quercetin significantly improved antioxidant capacity in laying hens, as shown by increased SOD activity and decreased malondialdehyde (MDA) levels. These findings agree with those reported by Liu *et al*. [14], who saw lower MDA levels and higher SOD activity in hens supplemented with quercetin. Similar antioxidant effects have also been reported in aging hens [22] and hens exposed to heat stress [21]. Lin *et al*. [17] further showed that mulberry leaf supplementation with quercetin increased SOD activity while also decreasing MDA levels in hen serum.

SOD is a crucial first-line antioxidant enzyme that safeguards cells against oxidative damage [60] and has an important role in ovarian development and reproductive function [61]. In contrast, MDA is a widely used biomarker of lipid peroxidation and oxidative stress in serum and tissues [62]. Although limited data prevented a meta-analysis of glutathione peroxidase (GSH-Px) and catalase (CAT) activities, the observed changes in SOD and MDA strongly indicate that quercetin supplementation effectively reduces oxidative stress by enhancing endogenous antioxidant defenses. Reduced oxidative damage likely contributed to the noted improvements in performance, productivity, and egg quality.

### Dose-dependent responses and optimal supplementation range

The current meta-analysis showed that the effects of quercetin on productivity and antioxidant status depend on the dose. Supplementation between 400–600 mg/kg most effectively improved LR, FER, and SOD activity. These results match those of El-Saadany *et al*. [12], who found maximum egg production, feed efficiency, and antioxidant activity in hens given 600 mg/kg of quercetin compared to lower or higher doses. Similarly, Liu *et al*. [14] noted that 500 mg/kg dietary quercetin increased laying performance and antioxidant defenses while decreasing lipid peroxidation.

Yang *et al*. [11] further reported that hens receiving 400 mg/kg quercetin had LR similar to those receiving 600 mg/kg, with better feed-to-egg efficiency at the lower dose. These findings suggest that moderate supplementation offers the best benefits while reducing potential inefficiencies or safety issues associated with higher doses. Based on the overall evidence, the safe and effective dietary range for quercetin supplementation in laying hens is 400–600 mg/kg.

### Study limitations and future research directions

Several limitations of this study should be acknowledged. First, significant heterogeneity was observed across all analyzed parameters, with I² values exceeding 75%, which could affect the accuracy of pooled effect estimates. Second, breed-specific subgroup analyses were not possible due to a lack of data for individual hen breeds. Third, most of the included studies (77.77%) were conducted in Asian countries, which may limit the applicability of the findings to other production systems and regions. Finally, this meta-analysis focused solely on quercetin as a single dietary intervention and did not assess its combined effects with other antioxidants, enzymes, vitamins, or minerals.

Future research should fill these gaps by conducting well-designed, multi-regional studies that investigate breed-specific responses and evaluate the synergistic effects of quercetin when combined with other functional feed additives.

## CONCLUSION

This meta-analysis provides comprehensive quantitative evidence that dietary quercetin supplementation positively affects productive performance, egg quality, metabolic health, and antioxidant defenses in laying hens. Pooled results showed significant improvements in LR, FER, EW, HU, ST, and YC, while feed intake remained unchanged. Quercetin supplementation also beneficially modulated blood metabolites by lowering SGPT, glucose, and total cholesterol levels, and increasing HDL levels, indicating improved liver function and lipid metabolism. Additionally, quercetin significantly reduced malondialdehyde levels and boosted SOD activity, reflecting stronger antioxidant defenses and less oxidative stress. Dose–response analyses revealed linear effects on metabolic parameters and quadratic responses for LR, feed efficiency, and antioxidant activity, with optimal results consistently seen at dietary quercetin levels of 400–600 mg/kg.

From a practical perspective, quercetin is a promising natural feed additive that can enhance productivity, egg quality, and physiological resilience in laying hens. Supplementing at 400–600 mg/kg can improve feed efficiency and laying performance while supporting metabolic and antioxidant health. This contributes to more sustainable and welfare-focused poultry production systems. The use of quercetin may also decrease reliance on synthetic growth promoters and align with One Health nutritional strategies.

The main strengths of this study include combining data from 27 independent studies using rigorous PRISMA-guided methodology and strong random-effects meta-analytical models. The use of subgroup and meta-regression analyses helped identify dose-dependent responses and important moderators such as treatment duration, hen age, and quercetin form. Significantly, this analysis evaluated productive, metabolic, and oxidative stress outcomes together, offering a comprehensive view of quercetin’s biological effects in laying hens.

Despite these strengths, several limitations must be recognized. High heterogeneity was observed across most outcomes, reflecting differences in experimental design, hen genotypes, environmental conditions, and supplementation protocols. Breed-specific effects could not be assessed due to limited data, and most studies were from Asia, which might limit their applicability globally. Additionally, the analysis only focused on quercetin as a single intervention and did not explore potential synergistic effects with other dietary antioxidants or nutrients.

Future studies should focus on well-controlled, multi-breed, and multi-regional trials to validate these findings across diverse production systems. Research should also investigate the combined use of quercetin with other bioactive compounds, enzymes, vitamins, or minerals to evaluate potential synergistic effects. Long-term studies on reproductive longevity, egg storage quality, and economic outcomes would further strengthen evidence-based recommendations.

In conclusion, dietary quercetin supplementation provides consistent, dose-dependent benefits for productivity, egg quality, metabolic health, and antioxidant status in laying hens. Supplementing with 400–600 mg/kg appears to be a safe and effective way to improve performance and physiological resilience, supporting the use of quercetin as a functional feed additive in sustainable poultry nutrition.

## DATA AVAILABILITY

The supplementary data can be made available from the corresponding author upon request.

## AUTHORS’ CONTRIBUTIONS

SH: Conception and design of the study, data collection, data analysis, and interpretation, and drafted and revised the manuscript. HPW: Data collection and drafted the manuscript. HK: Data collection, analysis, and interpretation. SS: Supervised the study and revised the manuscript. FRPH, AP, BH, RNH, DDL, RP, and AAY: Data collection and reviewed and revised the manuscript. JS and AJ: Critical review, interpretation, and manuscript revision. All authors have read and approved the final version of the manuscript.
